# Dendrimer-coated carbon nanotubes deliver dsRNA and increase the efficacy of gene knockdown in the red flour beetle *Tribolium castaneum*

**DOI:** 10.1038/s41598-020-69068-x

**Published:** 2020-07-24

**Authors:** Catriona H. Edwards, Craig R. Christie, Andrea Masotti, Antonella Celluzzi, Andrea Caporali, Ewan M. Campbell

**Affiliations:** 1 School of Biological Sciences, University of Aberdeen, Aberdeen, UK; 2 Research Laboratories, Bambino Gesù Children’s Hospital, Rome, Italy; 3 Centre for Cardiovascular Science, University of Edinburgh, Edinburgh, UK; 4 Centre for Genome Enabled Biology and Medicine, University of Aberdeen, 23 St. Machar Drive, Aberdeen, AB24 3RY UK

**Keywords:** Nanobiotechnology, RNAi, Animal physiology

## Abstract

In this study, the use of dendrimer-coated carbon nanotubes (CNTs) as a delivery vehicle for dsRNA was assessed in *Tribolium castaneum*. Exposure to low dosages of polyamidoamine dendrimer carbon nanotubes (PAMAM-CNTs) did not affect *T. castaneum* larval mortality. Expression of key apoptotic factors, Dronc (*Tc12580*), Dredd (*Tcn-like, Tc014026*) and Buffy, (*Tcinhib apop1*), which can act as toxicity indicators, were not altered in *T. castaneum* larvae following injection of PAMAM-CNTs. The level of knockdown of two target genes, α-tubulin and mitochondrial RNA polymerase (mtpol), were significantly increased when larvae were injected with double-stranded RNA bound to CNTs (PAMAM-CNT-dsRNA), compared to those injected with target dsRNA alone. PAMAM-CNTs were visualised in cellular vacuoles and in the cell nucleus. Increase occurrence of a blistered wing phenotype was found in a subset of PAMAM-CNT-dsRNA_αtub_ injected larvae, relative to the level seen in larvae injected with naked dsRNA_αtub_ alone. These results suggest that the use of functionalised CNTs for dsRNA delivery could increase the efficacy of RNA interference in insect pest species.

## Introduction

RNA interference (RNAi) is a promising tool for the control of insect pests. Introduction of exogenous double-stranded RNA (dsRNA) is effective at triggering gene knockdown and associated phenotypes in many problem pest species such as the Western Corn Rootworm^1^, the Colorado potato beetle^2^ and the Varroa mite^3^. New developments in dsRNA delivery methods to crop pests, such as dsRNA expression in transgenic plants^4^ and foliar application^5^, offer the possibility of dsRNA-based insecticides^6^. Despite this promise, there is notable variation in RNAi response between insect species^7^, with some, such as many lepidopterans^8^, unable to mount a strong RNAi response to dsRNA. Ingestion of dsRNA can be a valid route for delivery, yet many insects secrete enzymes in their gut that breakdown dsRNA before the latter can elicit an effect on target gene transcripts^9–11^. When dsRNA enters into the cytoplasm, it is generally very effective in mounting an RNAi response^12,13^. However, the route to the cytoplasm can act as a barrier to effective RNAi, with stability and uptake of dsRNA within the insect thought to be the significant limiting factor^10,11,14,15^. Therefore, the protection of dsRNA from degradation coupled with an efficient cellular uptake is key to RNAi success. The development of delivery vehicles or carriers that enable these two processes is now necessary to fully realise the potential for dsRNA as an effective control measure.

Combining dsRNA with other substances that act as efficient carriers, such as chitosan^16,17^, perfluorocarbon nanoparticles^18–20^ or ribonuceloparticles^21^, can stabilise dsRNA and increase the chances of successful gene knockdown. Encapsulation of dsRNA within carbon quantum dots^22^ and liposomes^23,24^ has also shown some promise in increasing efficacy in more challenging insect species.

In the last decade, carbon nanotubes (CNTs) have emerged as a novel and alternative delivery agent for different biomolecules, including siRNAs in the field of gene-silencing^25^. Together with the large surface area, the relatively low weight makes them ideal, particularly for applications in nanomedicine and drug delivery. However, due to their hydrophobic nature, pristine CNTs cannot be functionally integrated into biological systems unless they undergo surface functionalization to both allow their suspension and become more biocompatible^26^. The use of cationic molecules or polymers, such as polyamidoamine dendrimer (PAMAM), to improve CNTs functional properties leads to their electrostatic interaction with negatively charged siRNAs, thus increasing nucleic acid loading on nanomaterials^27^. Moreover, functionalization of CNTs with polymers is a key process to obtain a non-cytotoxic CNT-based delivery system^28^. Finally, functionalization of CNTs may also facilitate the efficiency of delivery allowing them to penetrate cell membranes directly or by endocytosis^29^. We have successfully used PAMAM-functionalized CNTs (PAMAM-CNTs) in previous studies to deliver miR-503 in primary endothelial cells and HeLa cells to regulate angiogenesis^30,31^.

*Tribolium castaneum* is an ideal model for RNAi. As well as being easy to rear in laboratory settings the species has a well-characterised genome^32^ and displays a robust systemic RNAi response^33^, which can even be spread to offspring^34^.

In this study, we used an abundant, well characterised and widely distributed housekeeping gene, α-tubulin (Tc_αTub1), as well as a previously characterised transcription accessory protein, mtpol, as targets for gene silencing. dsRNA will henceforth be referred to as dsRNA_αtub_ and dsRNA_mtpol_ respectively. We used PAMAM-CNTs as a delivery vehicle for dsRNAs in *T. castaneum* and showed that they increase the efficacy of RNAi and some phenotypic response, thus representing a safe, effective delivery vehicle for eliciting gene-silencing in economically important insect pests.

## Results

### Low doses of PAMAM-CNTs do not increase mortality of *T. castaneum* larvae

**Figure 1:**
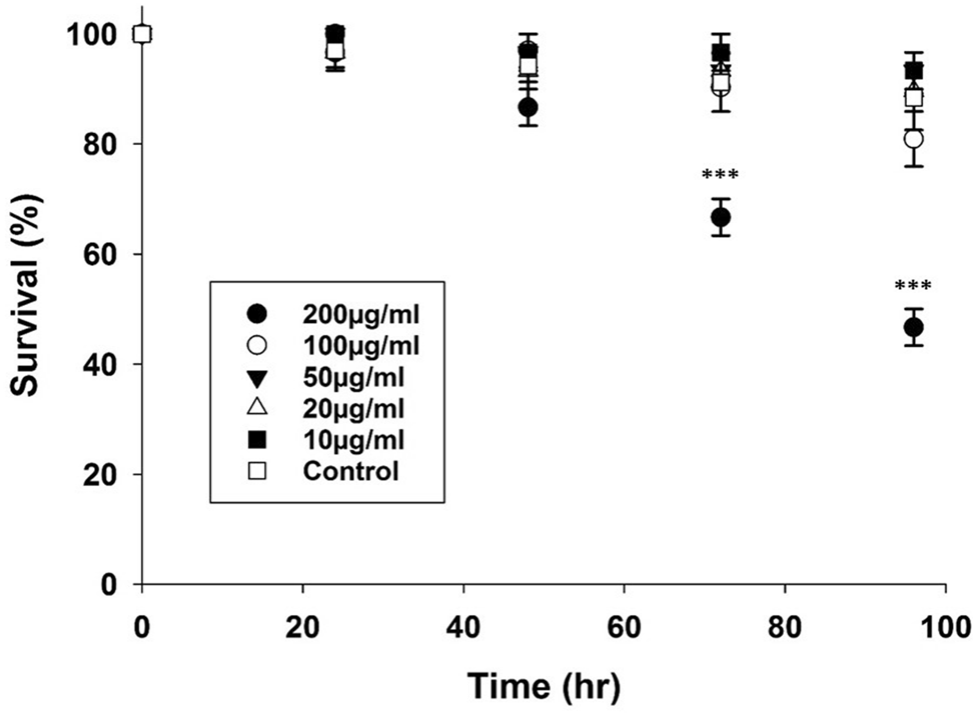
Survival of 4th instar *T. castaneum* larvae following injection with different dosages of PAMAM-CNTs (10–200 µg/mL) or control solution. Mortality was monitored every 24 h following microinjection. At 72 and 96 h post-injection, there was significantly increased mortality in groups injected with 200 µg/mL PAMAM-CNTs relative to controls (72 h, *P* = 0.029; 96 h, *P* = 0.008). No other dosages significantly impacted the survival of *T. castaneum* over the observed period. Data are means ± SEM. Three replicate groups per treatment (n = 9–13 larvae each).

To assess whether PAMAM-CNTs alone were harmful to *T. castaneum*, 4th instar larvae were micro-injected with PAMAM-CNTs ranging in concentration from 10–200 µg/mL or with control dye solution. No increased mortality was seen for any PAMAM-CNT dose compared to dye injected control larvae at 24 h (*P* = 0.851) or 48 h (*P* = 0.288) (Fig. 1). At 72 h and 96 h post-injection, mortality was significantly higher for *T. castaneum* injected with 200 µg/mL PAMAM-CNTs relative to dye-injected control groups (72 h 33.3% vs 8.8%, *P* = 0.029; 96 h 57% vs 8%, *P* = 0.008) (Fig. 1). No significant increase in mortality was observed following injection of other doses of PAMAM-CNTs, relative to dye-injected controls, indicating that doses of 100 µg/mL PAMAM-CNTs or lower are not lethal to *T. castaneum* larvae over this period. No visible abnormalities were observed in larvae in any of the groups throughout this trial. For all further experiments, a concentration of 50 µg/mL PAMAM-CNTs was used in microinjections.

### Cellular stress gene expression is not increased by PAMAM-CNTs

**Figure 2:**
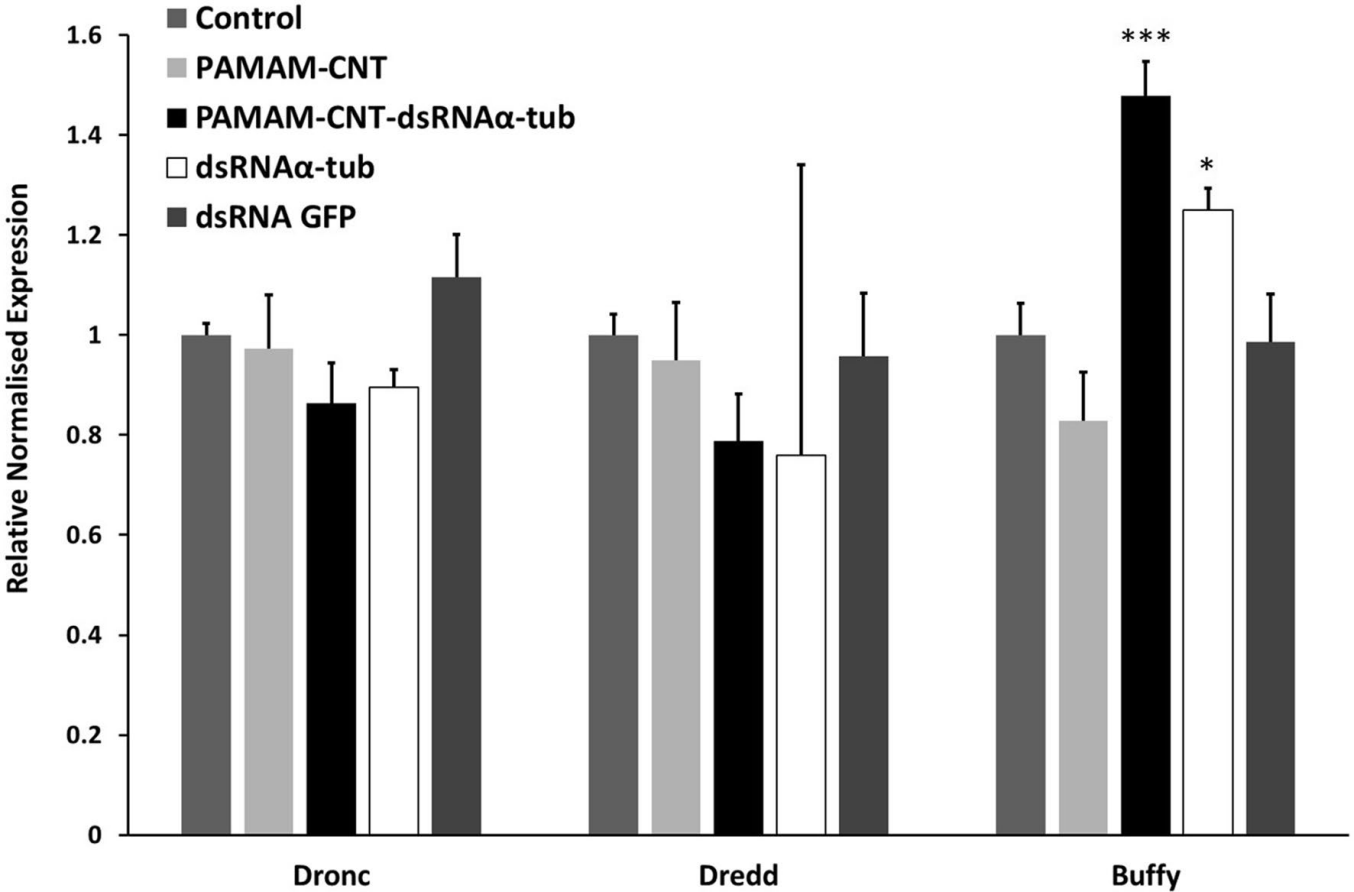
Relative transcript expression of three stress response genes in *T. castaneum* following micro-injection with PAMAM-CNTs and dsRNA. *T. castaneum* larvae were injected with 50 µg/mL naked PAMAM-CNTs or 50 µg/mL PAMAM-CNT-dsRNAαtub and expression measured at 96 h, along with control (saline), dsRNAαtub and dsRNA GFP. Asterisks represent significant differences between untreated and treated larvae (*P* < 0.001). The data are presented as means ± SEM of three replicate samples.

We have previously shown that PAMAM-CNTs at 50 µg/mL, equivalent to the dose used in the present study, have low cyto-toxicity^30,31^. Cellular viability of human umbilical vein endothelial cells (HUVECs) is not altered compared to controls in the presence of PAMAM-CNTs. However, in order to assess possible stress responses induced by PAMAM-CNTs in *T. castaneum*, qPCR was used to measure the expression of three target genes, *Dronc* (*Tc12580*), *Dredd* (*Tcn-like*, *Tc014026*) and *Buffy* (*Tcinhib apop1*), each involved in apoptosis and stress response, following exposure to both naked-PAMAM-CNTs alone and PAMAM-CNTs complexed with dsRNA_αtub_. No change in gene expression was seen in any of these stress response genes following exposure to PAMAM-CNTs alone (Fig. 2) or control dsRNA_GFP_.. PAMAM-CNT-dsRNA_αtub_ exposure did not significantly alter the expression of Dronc (*tc12580*) (*P* = 0.504) or Dredd (*Tcnlike*) (*P* = 0.256), but, interestingly, significantly increased the expression of Buffy (*Tcinhib apop1*) (*P* = 0.05), compared to levels in both control and PAMAM-CNT only injected larvae (*P* = 0.05). dsRNA_αtub_ alone also caused an increase in the expression of Buffy (*Tcinhib apop1*) compared to levels in both control and PAMAM-CNT only injected larvae (*P* < 0.05). This suggests that although the PAMAM-CNTs do not affect cellular stress, the silencing of α-tubulin may affect apoptotic pathways in *T. castaneum,* although this was not within the scope of this study and so remains speculative. Given its vital role in cellular architecture this is not an unexpected result and dovetails with the phenotype and mortality observed in further experiments.

### PAMAM-CNTs aggregate within *T. castaneum* cells

**Figure 3:**
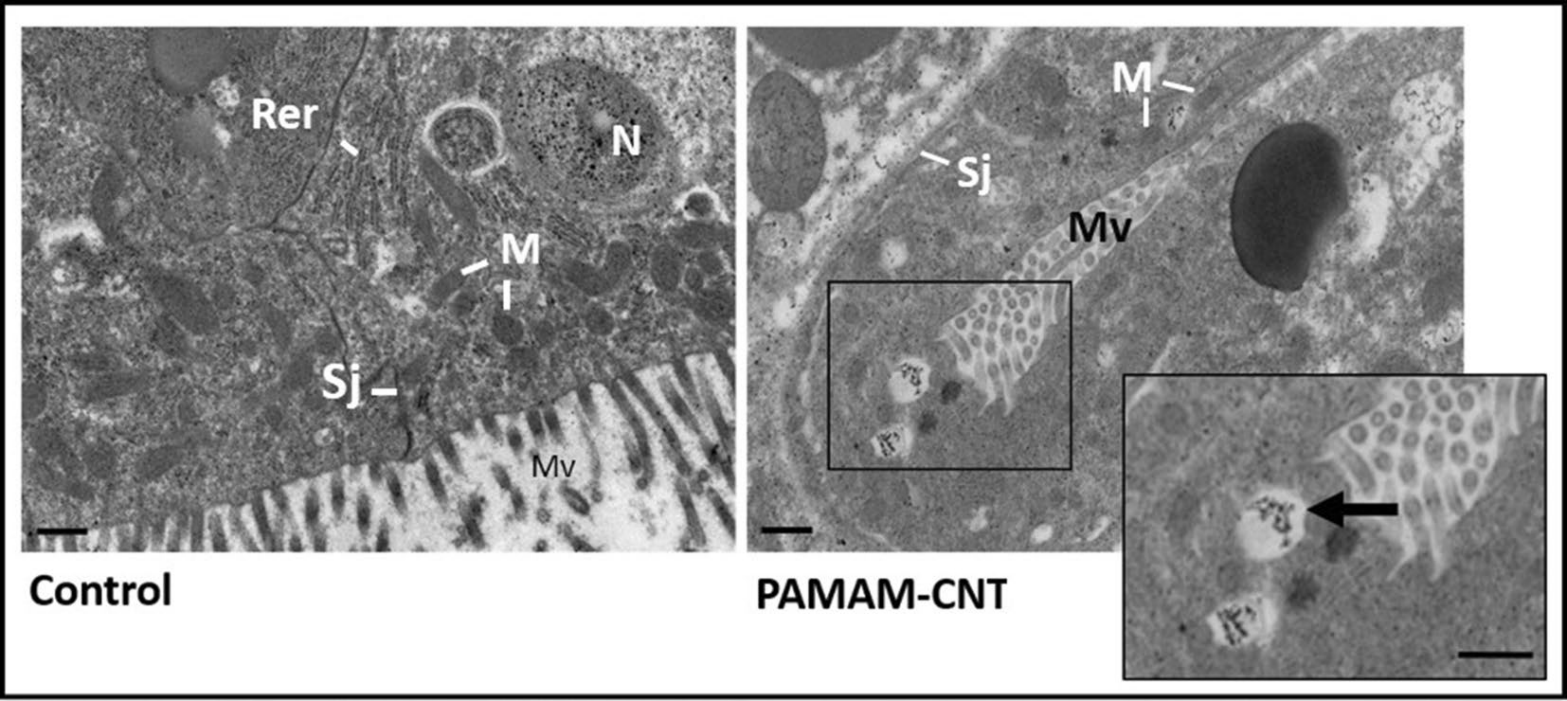
Transmission electron microscope images of sections of saline (control) and PAMAM-CNT-dsRNA_αtub_ exposed *T. castaneum* larvae. Representative sections proximal to midgut tissue are shown. Arrows highlight intracellular vesicles containing PAMAM-CNT aggregates. Labels are rough endoplasmic reticulum (Rer), septate junction (Sj), mitochondria (M), microvilli (Mv) and nucleus (N) Scale = 2 µm.

Transmission electron microscopy (TEM) on sections of *T. castaneum* larvae was used to confirm the intracellular localisation of PAMAM-CNT-dsRNA in different tissue types including in cells proximal to the midgut (Fig. 3). No visible cytotoxicity was observed in PAMAM-CNT-dsRNA-exposed *T. castaneum* cells when compared with saline-injected controls. We cannot visualise the dsRNA directly, but PAMAM-CNTs were visible within cells 48 h post-injection demonstrating that intra-haemocoelic inoculation results in an efficient uptake of PAMAM-CNT-dsRNA. PAMAM-CNTs were observed as aggregates within cells, in what appear to be intracellular vesicles, in agreement with previous findings^30^. PAMAM-CNTs were also apparent within cell nuclei and were only rarely observed individually in the cytoplasm.

### PAMAM-CNT-dsRNA complexes increase knockdown efficiency and phenotypic effects compared to naked dsRNA

**Figure 4:**
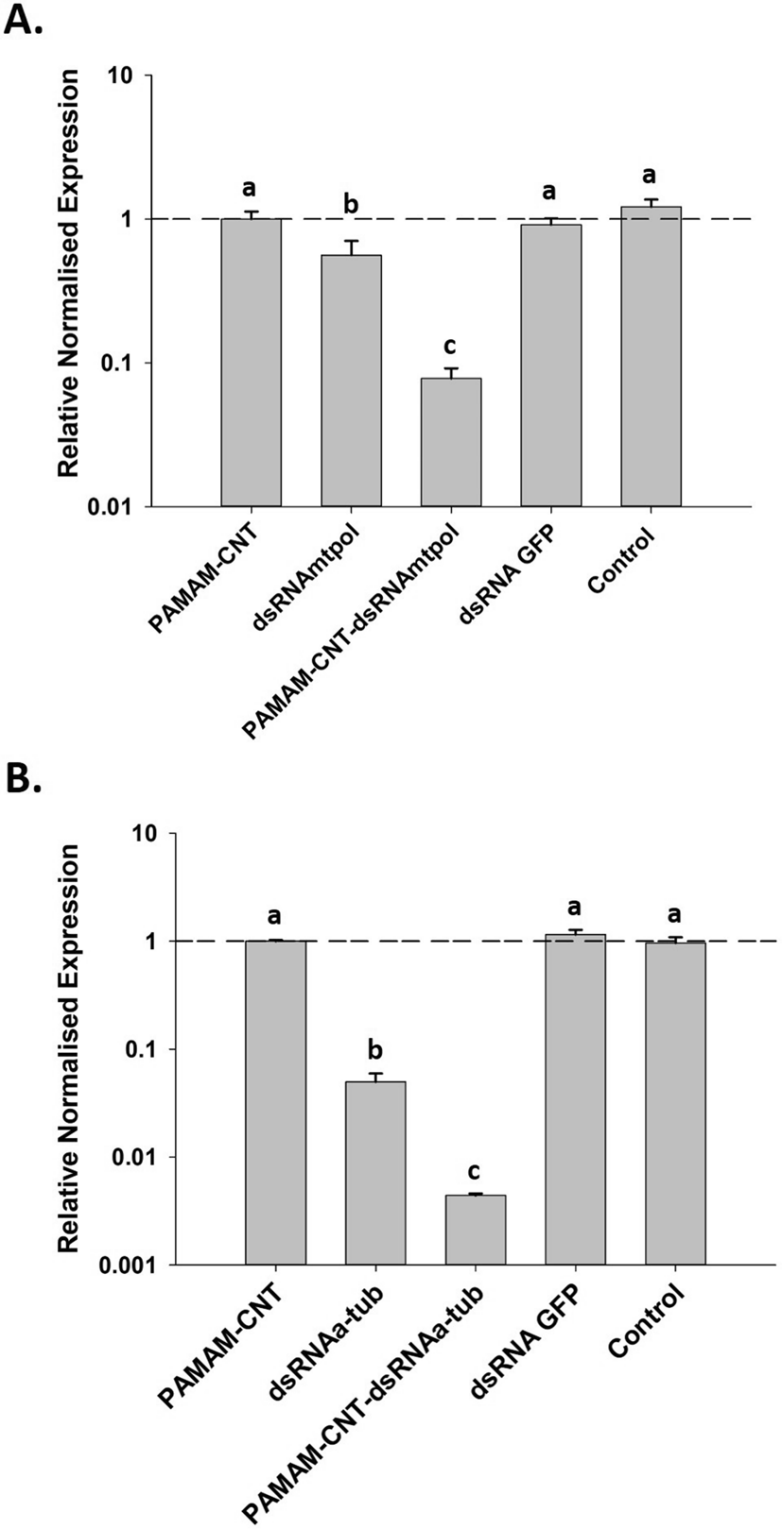
Relative normalised gene expression of targets after injection of PAMAM-CNTs, dsRNA or PAMAM-CNT-dsRNA complexes. mtpol expression (**A**) was significantly decreased following injection of naked dsRNA_mtpol_ at 72 h (*P* < 0.05) post-injection. mtpol expression was also significantly reduced in PAMAM-CNT-dsRNA_mtpol_ injected larvae at 72 h (*P* < 0.001). Expression was significantly lower in PAMAM-CNT-dsRNA_mtpol_ compared to dsRNA_mtpol_ alone at 72 h (*P* < 0.01). α-tubulin expression (**B**) was significantly decreased following injection of naked dsRNA_αtub_ (*P* < 0.001). The α-tubulin expression was significantly reduced in PAMAM-CNT-dsRNA_αtub_ injected larvae (*P* < 0.001). Expression was significantly greater in PAMAM-CNT-dsRNA_αtub_ compared to dsRNA_αtub_ alone (*P* < 0.001). Data are presented as means ± SEM (n = 4 per treatment) as Log_10_ relative normalised expression. Labelling with different letters indicates significant difference between treatments (a > b > c).

To evaluate if dsRNA complexed with functionalised CNTs is effective in gene silencing, we targeted α-tubulin 1^35^ (αtub: XP_966492) and DNA-directed RNA polymerase (mtpol: XM_962507.3) in *T. castaneum* by microinjection of beetle larvae. The expression of *mtpol* was reduced significantly by both naked dsRNA_mtpol_ and PAMAM-CNT-dsRNA_mtpol_ compared to control injections (Fig. 4a). At 72 h larvae injected with naked dsRNA_mtpol_ compared to control had a 43% reduction in expression compared to controls (*P* < 0.05) whereas the decrease in *mtpol* expression was more significant in PAMAM-CNT-dsRNA_mtpol_ injected larvae (93% reduction, *P* < 0.001) There were notable differences in knockdown levels over time (Fig. S1). Significant reduction in gene expression was seen 48 h post-injection by both naked dsRNA_mtpol_ (87%, *P* < 0.001) and PAMAM-CNT-dsRNA_mtpol_ (89%, *P* < 0.001). At 96 h larvae injected with naked dsRNA_mtpol_ showed complete recovery of expression to baseline levels whereas larvae injected with PAMAM-CNT-dsRNA_mtpol_ continued to have reduced expression levels (32%, *P* < 0.05). The expression of *α-tubulin* was investigated at 72 h and was significantly decreased following injection of naked dsRNA_αtub_ (Fig. 4b) (95% or 20 fold reduction in expression, *P* < 0.001). The decrease in *α-tubulin* was significantly higher in PAMAM-CNT-dsRNA_αtub_ injected larvae (99.5% or 226 fold reduction, *P* < 0.001) (Fig. 4b). This indicates that PAMAM-CNT-dsRNA complexes can significantly improve knockdown efficacy compared to naked dsRNA alone even at lower concentrations. Control dsRNA_GFP_ or saline controls did not affect target transcript levels significantly.

**Figure 5:**
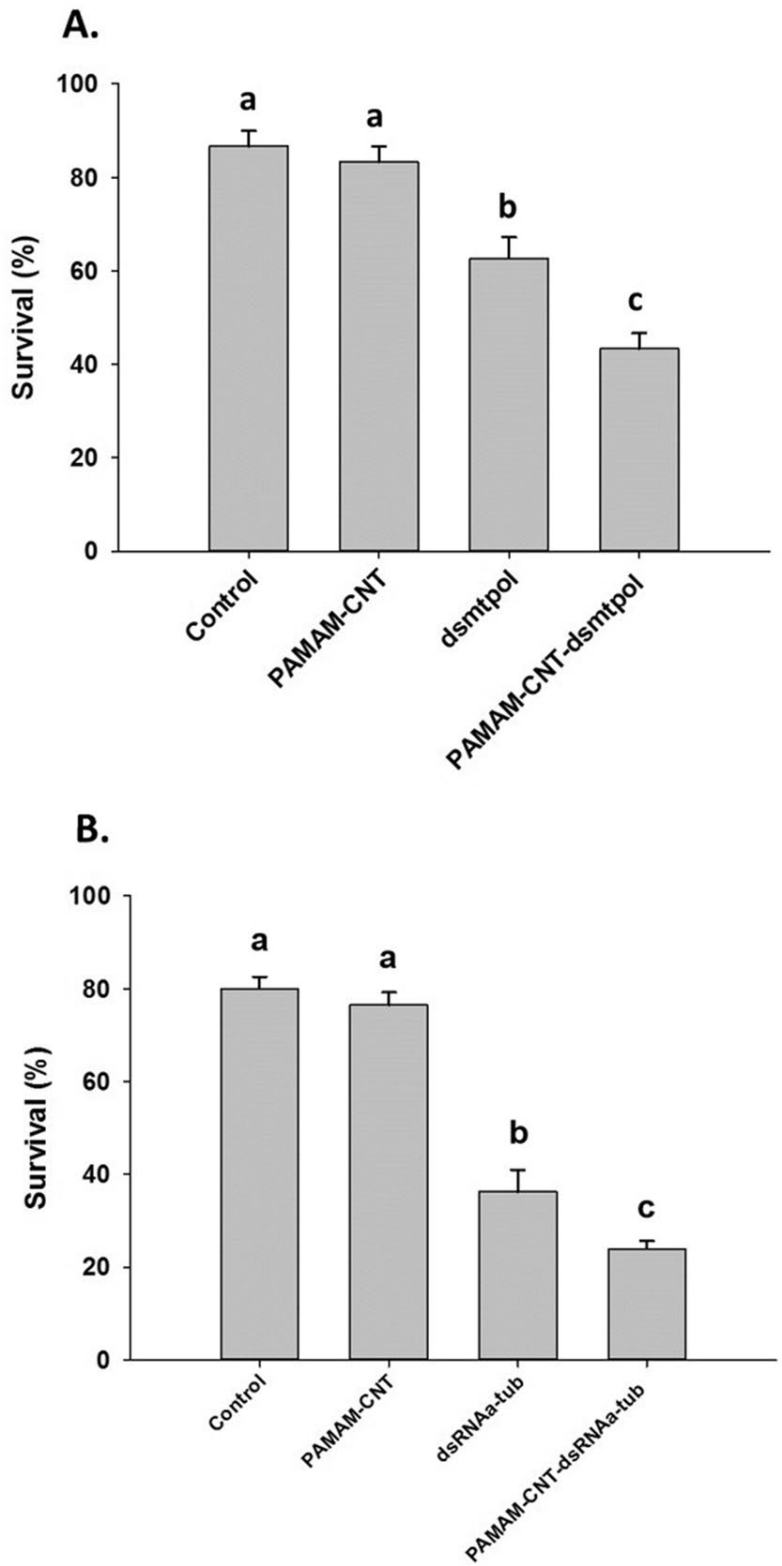
Survival of larvae injected with saline (control), naked PAMAM-CNTs, dsRNA or PAMAM-CNT-dsRNA at 96 h post-injection. Larvae were assessed every 24 h for mortality. PAMAM-CNT-dsRNA_mtpol_ injected larvae (**A**) had significantly increased mortality compared with dsRNA_mtpol_ alone (*P* < 0.05). PAMAM-CNT-dsRNA_αtub_ injected larvae (**B**) had significantly increased mortality compared with dsRNA_αtub_ alone (*P* = 0.041). The data are presented as means ± SEM (4 replicate groups, n = 10–11 per group). Different letters indicate significant difference in survival between treatments (a > b > c).

**Figure 6:**
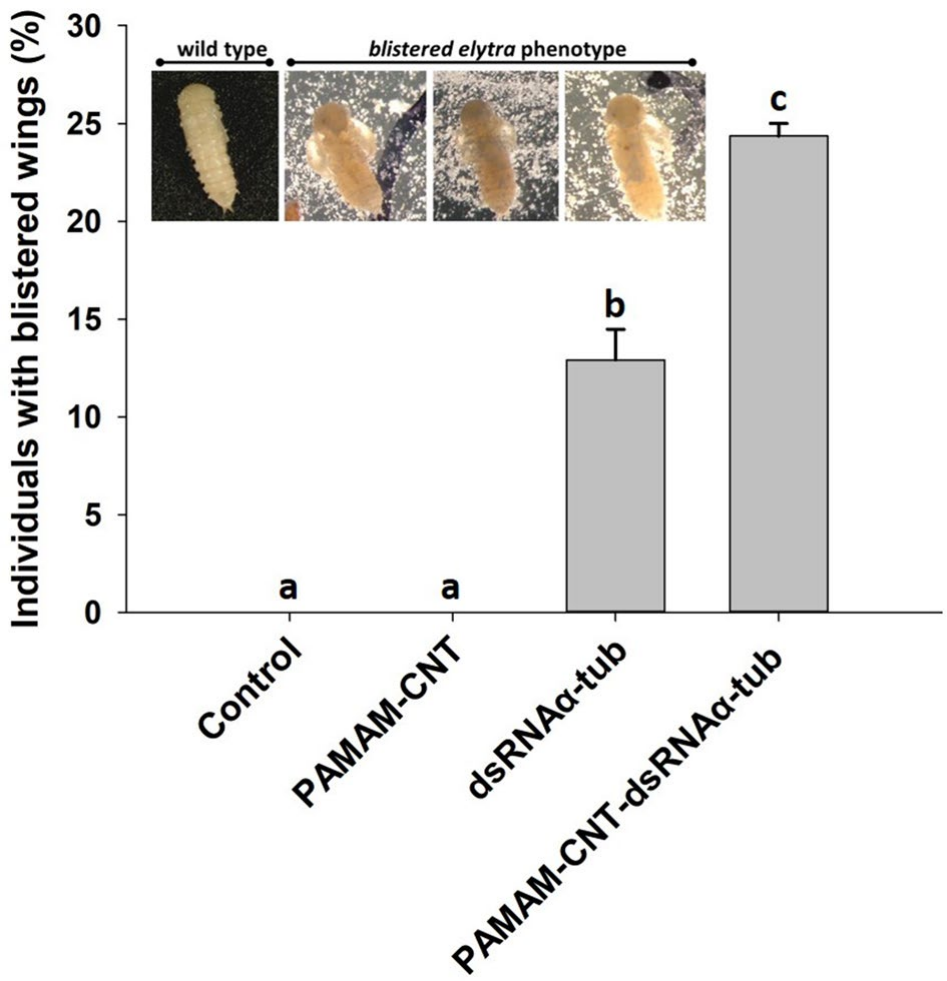
Frequency of blistered wing phenotype in *T. castaneum* pupae. No phenotype was seen in *T. castaneum* larvae injected with saline (control) or naked PAMAM-CNTs after pupation. A blistered phenotype was observed, post-pupation, in larvae injected with dsRNA_αtub_ or PAMAM-CNT-dsRNA_αtub_ exposed. The phenotype was significantly more frequent in the PAMAM-CNT-dsRNA_αtub_ injected group compared to dsRNA_αtub_ alone (*P* = 0.016). The data are presented as means ± SEM (n = 41 per treatment). Different letters indicate a significant difference in survival between treatments (a > b > c).

As well as measurable knockdown of two target genes, the injection of target dsRNA and PAMAM-CNT-dsRNA significantly decreased survival of *T. castaneum* larvae, relative to larvae injected with saline or PAMAM-CNTs alone (Fig. 5). Again, the effect seen was greater for PAMAM-CNT-dsRNA injected larvae, compared to those injected with both dsRNA_mtpol_ (*P* = 0.04) (Fig. 5a) or dsRNA_αtub_ (*P* = 0.041) (Fig. 5b). In some individuals a blistered phenotype in the early wing structure was seen in pupal development after larvae were exposed to dsRNA_αtub_ or PAMAM-CNT-dsRNA_αtub_, which was not seen in control or naked PAMAM-CNT injected *T. castaneum* (Fig. 6). The blistered wing phenotype was observed significantly more frequently in surviving cohorts of *T. castaneum* injected with PAMAM-CNT-dsRNA_αtub_ than in those injected with only dsRNA_αtub_ (24.3% vs 12.3%, *P* = 0.016). No abnormality in external morphology was observed in either PAMAM-CNT-dsRNA_mtpol_ or dsRNA_mtpol_ injected individuals.

## Discussion

RNAi has proven an effective means to silence targeted genes in a range of pest arthropod species^3,23,36^. This has allowed more detailed research into physiological pathways and molecular mechanisms such as reproductive behaviour and immune response^37,38^.

More promising still is the prospect of use RNAi directly as a pest control method, by targeting dsRNA toward vital targets that cause lethality when knocked down^37^. While RNAi has shown promise for the control and management of agricultural, veterinary and medically important pests^38^, many challenges of employing RNAi in real-world conditions still remain. Major challenges to overcome in insect pests include the variable response of different insect orders to RNAi^8^, the rapid clearance and degradation of ingested dsRNA^39,40^ and inefficient uptake of dsRNA into cells.

To realise RNAi as an effective tool in managing agricultural pests (see^38^ for review^38^), medical and veterinary treatments, there is a pressing need to increase dsRNA stability, to ensure delivery to cells wherein the effector can trigger a response^30^. In some insect species, naked dsRNA is rapidly degraded in the saliva and midgut, preventing dsRNA from entering cells and triggering an exogenous RNAi response^12,13^. Suitable carriers that can protect dsRNA en route to target cells are required to enable efficient gene knockdown in such species.

A range of different carriers has been proven to be effective facilitators for delivery and stabilisation of both drugs and RNAi effectors including chitosan^16,17^, nanoparticles^18–20^, ribonucleoparticles^21^, carbon quantum dots^22^ and liposomes^23,24^. Nanomaterials are particularly attractive as carriers for the delivery of RNAi effectors^38,41^. In previous works, we have demonstrated that multi-walled carbon nanotubes are effective means to deliver miRNAs into human umbilical vein endothelial cells (HUVECs)^30^ or HeLa cells^31^. The polyamidoamine dendrimer (PAMAM) used in our study allows greater electrostatic interaction with nucleic acids, in this case, dsRNA, thus increases the loading onto CNTs^30^. In the present study, we show that functionalised CNTs have a low toxicity profile in *T. castaneum*, are delivered into the cytoplasm and, when used to carry dsRNA, trigger a higher level of gene silencing and phenotypic response than naked dsRNA alone.

The RNAi response in *T. castaneum* is one of the strongest and sustained in insects tested to date^42^. RNAi in both adult and larva beetles is systemic and can be triggered by injection of dsRNA in any tissue or stage^43^. Despite this already substantial reaction, we demonstrated that the effect of RNAi can be increased further by employing a nanostructured delivery agent. We showed that PAMAM-CNT, when complexed with dsRNA targeting mtpol and injected at equivalent dsRNA concentrations into larvae, showed a significantly increased efficacy. When targeted against the highly abundant housekeeping gene α-tubulin, coupled to PAMAM-CNT the knockdown is significant even when injected at a significantly lower concentration than the naked dsRNA_a-tub._ An increase in efficacy in *T. castaneum* could translate to even greater effectiveness if PAMAM-CNT coupled dsRNA is used in species that show a low or ineffective response to dsRNA alone. Knockdown of both mtpol and α-tubulin, as measured by gene expression, was increased significantly by PAMAM-CNT coupled dsRNA. Gene expression reduction in both targets correlated with an increase in the mortality of larvae and also, in the case of α-tubulin knockdown, with the number of blistered wing phenotypes in surviving larva that underwent metamorphosis to pupa. An RNAi screen has previously knocked down ~ 33% of predicted *T. castaneum* genes, with functional screens in fifth-instar larvae and during embryogenesis by parental RNAi^42^. Since α-tubulin, was not investigated in this screen, further investigations would need to be undertaken to confirm the efficacy of this phenotypic response. Curiously the response was not seen in all individuals which may reflect that it is not a stable or consistent phenotype. In *C. elegans* it has been shown that tubulin has some redundancy^44^. As there are 12 tubulin family members in the Tribolium genome, there could also be redundancy mechanisms at play in the current studyand that knockdown of Tc_αTub1, may either have off-target effects on wing development physiology or, more likely, that another α-tubulin may restore normal development through redundancy mechanisms. In silico analysis between *Tribolium* α-tubulins shows no identical stretches of 19 nucleotides to our target dsRNA necessary for triggering gene knockdown, the longest stretch was a single run of 15 nucleotides with Tc_αTub2.

We demonstrated that PAMAM-CNTs have a very low toxicity profile to *T. castaneum.* No increases in mortality were seen for any dose of PAMAM-CNTs in two days following exposure. After this period, only larvae exposed to the highest dose of PAMAM-CNTs had marginally increased mortality. PAMAM-CNTs displayed low cytotoxicity in HUVECs at levels similar to the doses used in the current study^30^ but toxicity of these kind of compounds has not been previously tested in insects. Toxicity of other classes of CNTs has been studied in *Drosophila melanogaster* embryos, where the accumulation of CNTs does not interfere with cellular divisions or embryonic development^45^. Adult *D. melanogaster*, when dusted, or larva, when fed with CNTs, have no increase in mortality compared to control groups^46^. In *D. melanogaster* somatic cells, functionalised CNTs demonstrate low cytotoxicity^47^. The soil arthropod, *Folsomia candida*, when exposed to CNTs show no detrimental effects on mortality or reproduction^48^. Intrahaemocoelic injection of CNTs, as carried out in the current study, resulted in the distribution of PAMAM-CNTs in cells 48 h after exposure, yet toxicity was extremely low. This evidence suggests CNTs, including PAMAM-CNTs, are an excellent and low toxic choice as a delivery effector for RNAi, further testing of oral or other delivery systems would be useful in future studies.

As well as larva mortality, we investigated the cellular stress response to PAMAM-CNTs by looking at the expression of three genes with homologues in *D. melanogaster* apoptotic stress models. In *D. melanogaster*, homologues of initiator caspases *Tc12580 *(Dronc) and *Tcn-like*/*Tc014026* (Dredd) show increased gene expression under cellular stress^49^, while a homologue of *Tcinhib apop1/Tc01380,* called Buffy*,* shows decreased gene expression in response to cellular stress^50^. Vecchio et al.^51^ showed that nano-materials can induce apoptotic stress responses in Drosophila as measured by increases in the initiator caspase *Dronc.* In the current study, we found no change in expression of the Dronc homologue *Tc12580* or two other apoptotic pathway transcript homologues *Tc014026* (Dredd) or *Tc01380* (Buffy) when treated with PAMAM-CNTs alone, indicating there is no induction of cellular apoptosis by PAMAM-CNTs in *T. castaneum* beetles at the concentrations used.

Utilising gene-silencing as a pest control strategy depends on the effector, in this case dsRNA, reaching a cellular localisation where it can trigger the endogenous RNAi response. The localisation of CNTs depend in turn on the method by which entry is gained to the cells. In HUVEC cells PAMAM-CNTs showed a superior transfection than other CNTs^30^. Previous studies have shown nanotubes are visible within endosomes in cells after only 30 min post-exposure^52^ and have been observed at 48 h post-transfection^30^. We have demonstrated, by TEM, that PAMAM-CNTs are similarly localised within vacuoles inside the cytoplasm of cells in a similar timeframe. In mammalian cells both CNTs in the cytosol and endosomes can be recruited into lysosomes over time and excreted by exocytosis. Another exit mechanism has been reported in polynuclear neutrophils and macrophages where nanotubes undertake slow biodegredation^53^. Tracking of functionalised CNTs in single HeLa cells show that there is active endocytosis of nanotubes into cells where they subsequently avoid degradation in cellular compartments such as Golgi apparatus^54^. It is possible, therefore, that PAMAM-CNT-dsRNA is also taken up by an endocytotic pathway, avoid degradation and thus increase the effect of RNAi as observed in the current study. We observed PAMAM-CNTs in the nucleus of cells. This supports experimental observations, and the model proposed by Mu et al^55^, whereby endocytosis of CNTs is followed by endosomal leakage and subsequent nuclear translocation.

## Conclusion

Our data indicated that CNTs functionalised with PAMAM are an effective delivery agent for gene specific dsRNA. PAMAM-CNT-dsRNA complexes can readily and rapidly enter target organism cells and elicit a superior RNAi effect than dsRNA alone. This enhanced modulation of gene expression in both targets results in higher larval mortality. When targeted against the vital cell structural protein α-tubulin it also results in a phenotypic response in some individuals. PAMAM-CNTs alone exhibited no toxic effects as measured by larval mortality or apoptotic stress response at a dose that was effective for a high RNAi response. PAMAM-CNTs are an effective delivery agent for dsRNA in *T. castaneum* beetles.

## Methods

### Preparation of dsRNA

Total RNA was extracted from 5 pooled late-larval *T. castaneum* using 1 mL Tri-reagent (Sigma-Aldrich Co. Ltd, Gillingham, UK), as per manufacturer's instruction. RNA was co-precipitated with 1.5 μL glycogen blue (Invitrogen Ltd, Paisley UK) and 5 μL 3 M sodium acetate in 95% ethanol and re-suspended in 100 μL of RNAse/DNAse-free molecular grade water.

After isolation, 1 μg total RNA was DNase treated with 1 μL (2U) RQ1-DNase (Promega UK Ltd, Southampton, UK) and 1 μL RQ1 buffer and incubated at 37 °C for 30 min. DNase-treated total RNA was reverse transcribed with iScript cDNA synthesis kit following manufacturers protocol (Bio-Rad Lab. Ltd, Watford, UK).

We used an abundant, well characterised and widely distributed housekeeping gene, α-tubulin (Tc_αTub1), as well as a previously characterised transcription accessory protein mtpol as targets for gene silencing. dsRNA will henceforth be referred to as dsRNA_αtub_ and dsRNA_mtpol_ respectively. *T. castaneum* specific dsRNA and negative control GFP-dsRNA were prepared with BLOCK-iT RNAi TOPO T7 transcription kits (Invitrogen Ltd, Paisley, UK), according to the manufacturer's instructions. Briefly, PCR was carried out using late larval stage *T. castaneum* cDNA in conjunction with Tcα-tub or Tcmtpol specific primers (Tc-dsRNA_αtub_, Tc-dsRNA_mtpol_, Table 1) or with control GFP plasmid (GFP: L4440, Addgene Inc., Cambridge, MA, USA) and GFP specific primers ^16^ (dsGFP, Table 1). PCR was carried out using the following cycling conditions: 1 cycle of 5 min at 94 °C, followed by 35 cycles of 1 min at 94 °C, 1 min at 58 °C and 45 s at 72 °C, followed by a final extension time of 15 min at 72 °C.

**Table 1:** Oligos for dsRNA preparation and target transcript evaluation.

Transcript	Function	NCBI ID	Oligo sequence	Size (bp)	Tm (°C)
*α-tubulin*	Cytoskeletal microtubule component	XP_966492	Tc-dsRNA_αtub__F: CAAGGAAATCGTCGACTTGG Tc-dsRNA_αtub__R: TGAAGGCACAGTCAGAATGC Tc-qPCR__atub__F: AAGACGCCGCCAATAACTAC Tc-qPCR__atub__R: TCGGCCAATTTACGGATG	275 91	51.8 51.8
*mtpol*	Mitochondrial RNA polymerase	XM_962507.1	Tc-dsRNAmtpo_F: GAATTACTCGACACAATCC Tc-dsRNAmtpo_R: CTGATTGTGGAAGATGAGG Tc-qPCR_mtpo_F: ATGTGCTCCACCTTGCAAAC Tc-qPCR_mtpol_R: TCAAATCCTTGCCTCTGGT	219 95	56.3 58.5
*Tc12580, Dronc*	Caspase initiator	NM_001170644.1	F: TCGGAGAAGGAGTACAAG R: TATCCACTCTTCCTCCAC	126	50.9
*TcNc-like*, *Dredd*	Caspase initiator	XM_015979881.1	F: ACTCCACTGACACCATAGAC R: GGACATAACTTGGTCCAC	77	51.4
*Tcinhib apop1, Buffy*	Apoptotic inhibitor	XM_961548.4	F: AACTGTTGAGGTGTGAGC R: CTATCCTCCAACAACTCC	103	51.0
GFP	Green fluorescent protein	MN114103.1	F: CCATCTAATTCAACAAGAATTGGGAC R: GGTCCTTCTTGAGTTTGTAAC	796	51.8
RPS3	Ribosomal protein of *T. castaneum*	XM_008194244.2	F: ACCTCGATACACCATAGCAAGC R: ACCGTCGTATTCGTGAATTGAC	60	50.5

Tm is annealing temperature of oligos.

Products were resolved on an agarose gel, excised and purified using a Qiagen gel extraction kit (Qiagen Ltd, Manchester, UK). TOPO-T7 linker was ligated to α-tub, mtpol and GFP reactions before a secondary PCR was carried out to produce sense and antisense templates. T7-RNA polymerase was used in transcription reactions with target templates to generate sense and antisense RNA. Single RNA strands were annealed and the resultant dsRNA purified and quantified by a ND-1000 Nanodrop Spectrophotometer (Labtech Inc., East Sussex, UK). Size was confirmed on denaturing gel using millennium RNA marker (Invitrogen Ltd, Paisley, UK) for size comparisons. dsRNA was ethanol precipitated and re-suspended in DEPC-treated water to a working concentration of 2.5 μg/μL and stored at − 80 °C prior to use.

### Preparation of CNTs and functionalization

Multi-walled carbon nanotubes (CNTs) were purchased from He Ji Co. Ltd. (Hong Kong; cat.no. M2704), polyamidoamine dendrimer generation 5 (PAMAM G = 5, cat.no. 536709) were purchased from Sigma-Aldrich Co. Ltd (Gillingham, UK) and used as received. For coating, CNTs (10 mg) were dispersed in distilled water (1.5 mL) and PAMAM (500 µL pure product) were added dropwise. Suspensions were placed in a sonicating water bath for 30 min and gently stirred overnight at room temperature. Samples were centrifuged at 20,800 xg for 30 min and washed with distilled water three times to remove the unbound polymers. The remaining solid was suspended in distilled water (1 mL) and sonicated for 2 min prior to further use. 2 µg of each target dsRNA were incubated with polyamine-coated CNTs at the weight ratio of CNTs/oligonucleotides 10:1 w/w corresponding to 20 µg of CNTs for 15 min at room temperature to allow complex formation. At this ratio, there is a full complex formation between dsRNA and PAMAM-CNTs^30,31^.

### Micro-injections for mortality and toxicity assessment

*T. castaneum* larvae were size and age sorted and held in flour/bran media at 28 °C/80% RH prior to trials (adapted from Linz et al^43^). Larvae were restrained using strips of adhesive tape at the anterior and posterior ends and microinjected in the dorsal side of the abdominal segments using heat-pulled micro-capillary borate silicon needles with a bore size of ~ 25 µm in conjunction with a Nanoject II system (Harvard Apparatus, USA).

Three replicate groups (n = 9–13 each) of larvae per treatment were injected with 120 nL PAMAM-CNTs. Five doses were used for toxicity assessment as well as saline injected control treatment groups. Non-injected control groups were taken from the same population at this time and maintained in the same environment. After injection larvae were removed from restraints and placed back into bran/flour in environmental chambers to recover. Larvae were monitored for mortality every 24 h by visual inspection. Dead larvae were removed from treatment groups. Statistical analysis was carried out using Minitab software (v.18) using ANOVA followed with Tukey comparison where necessary.

### Knockdown of target and assessment of apoptotic transcripts by quantitative RT-PCR

The ability of PAMAM-CNT-dsRNA to trigger gene silencing was assessed compared to naked dsRNA. 4th instar larvae (n = 30–32) were restrained and injected, as above, with 120 nL of 0.9% saline (control), naked PAMAM-CNT (50 µg/mL) and PAMAM-CNT-dsRNA_mtpol_ (50 µg/mL, effective 5 ng/µL dsRNA_mtpol_) or dsRNA_mtpol_ alone (5 ng/µL). For α-tubulin, larvae (n = 39–41 per treatment) were restrained and injected, as above, with 120 nL of 0.9% saline (control), naked PAMAM-CNT (50 µg/mL) and PAMAM-CNT-dsRNA_αtub_ (50 µg/mL, effective 5 ng/µL dsRNA_αtub_) or dsRNA_αtub_ alone (0.5 µg/µL). Larvae were monitored every 24 h for mortality and presence of overt phenotypic responses during pupation. At 96 h, a subset of larvae (n = 4, per treatment) were removed to assess gene knockdown.

RNA was extracted from individual larvae using Tri-reagent as described above. Two hundred ng RNA was reverse-transcribed using iScript cDNA synthesis kit for each sample (Biorad, Hemel Hempstead, UK). Resultant cDNA was quantified using a Nanodrop ND-1000 and the concentration adjusted to 5 ng/μL with RNase-free water.

Quantitative RT-PCR was performed on a CFX96 Real-Time PCR Detection system using iTaq universal SYBR® Green supermix (Bio-Rad, UK). Primers outside the targeted dsRNA regions were used in qPCR to measure knockdown of target gene transcript. Reactions were run in triplicate 20 μL volumes consisting of 10 μL iTaq supermix (BioRad), 4 μL water, 5 μL (5 ng/μL) of template cDNA and 1 μL (2 mM) respective primers. qPCR cycling conditions were as follows: 1 cycle of 3 min at 95 °C, followed by 35 cycles of 10 s at 94 °C and 30 s at the primer-specific annealing temperature (Table 1). Toxicity was assessed at the cellular level by the effect of CNTs on the gene expression of stress-mediated apoptotic response targets, *Tc13580*, *TcNc-like* and *Tcinhib apop1* also by qPCR with gene-specific primers (Table 1). The effect of CNTs as effective delivery vehicle of dsRNA was assessed by α-tubulin and mtpol expression.

Melting curve analysis was performed by incremental increases of 0.5 °C in 5 s from 65 to 95 °C. Duplicate control reactions with primer and template free reaction mixtures were included. A serial dilution of total combined cDNA pools was used to obtain standard curves and the corresponding primer amplification efficiency for each gene calculated. Cq values were extracted from CFX Manager software and analysis of melting curves was performed to confirm correct profiles for each gene transcript reaction. Efficiency was calculated from a standard serial dilution curve utilising CFX manager software. Relative normalised expression and significance was calculated by CFX manager utilising the delta delta ct method relative to reference gene RPS3^56^ (Table 1).

### Detection of PAMAM-CNT complexes within cells by TEM

Penetration of PAMAM-CNTs into larvae cells was assessed by TEM. Larvae were restrained and injected, as above, with 120 nL of 0.9% saline (control), PAMAM-CNT (50 µg/mL) and PAMAM-CNT-dsRNA_αtub_ (50 µg/mL) (n = 5 per treatment). At 48 h larvae were removed and fixed with 2.5% (v/v) glutaraldehyde in 0.1 M Sorensens buffer (pH 7.4), primarily by 5 µL injection and then by whole body immersion. Samples were post-fixed in 1% Osmium Tetroxide in water for 1 h and then dehydrated in ethanol series (30, 50, 75 and 100%) infiltrated and embedded in Spurrs resin. Ultrathin sections of 100 nm were cut using a Leica UC6 ultramicrotome. Sections were viewed in a JEOL 1,400 plus TEM at 80KV and images taken using an AMt UltraView camera.

### Phenotypic observation after exposure to PAMAM-CNT-dsRNA

Four replicate groups (n = 10–11 each) of larvae per treatment were restrained and injected, as above, with 120 nL of 0.9% saline (control), PAMAM-CNT (50 µg/mL), PAMAM-CNT-dsRNA_αtub_ (50 µg/mL, effective 5 ng/µL dsRNA_αtub_) or dsRNA_αtub_ alone (0.5 µg/µL). After injection larvae were removed from restraints and placed back into bran/flour in environmental chambers to recover. Larvae were left until after pupation and external morphology was observed. Dead larvae were removed from treatment groups. Statistical analysis was carried out using Minitab software (v.18) using ANOVA followed with Tukey comparisons.

## Supplementary information

Supplementary Information 1.
