# Supplementary material for: Dendrimer-coated carbon nanotubes deliver dsRNA and increase the efficacy of gene knockdown in the red flour beetle Tribolium castaneum
Source: Sci Rep. 2020 Jul 24;10:12422. doi: 10.1038/s41598-020-69068-x (PMC7381663; doi:10.1038/s41598-020-69068-x)
Supplement: Supplementary file 1 — Supplementary Information 1. [file 41598_2020_69068_MOESM1_ESM.pdf]

Dendrimer-coated carbon nanotubes deliver dsRNA and increase the efficacy of gene knockdown in the red flour beetle *Tribolium castaneum*

Catriona H Edwards[1], Craig R Christie[1], Andrea Masotti[2], Antonella Celluzzi[2], Andrea Caporali[3], Ewan M Campbell[4*]

[1]School of Biological Sciences, University of Aberdeen, Aberdeen, UK

[2] Bambino Gesù Children's Hospital, Research Laboratories, Rome, Italy

[3]Centre for Cardiovascular Science, University of Edinburgh, Edinburgh, UK

[4]Centre for Genome Enabled Biology and Medicine, University of Aberdeen, Aberdeen, UK

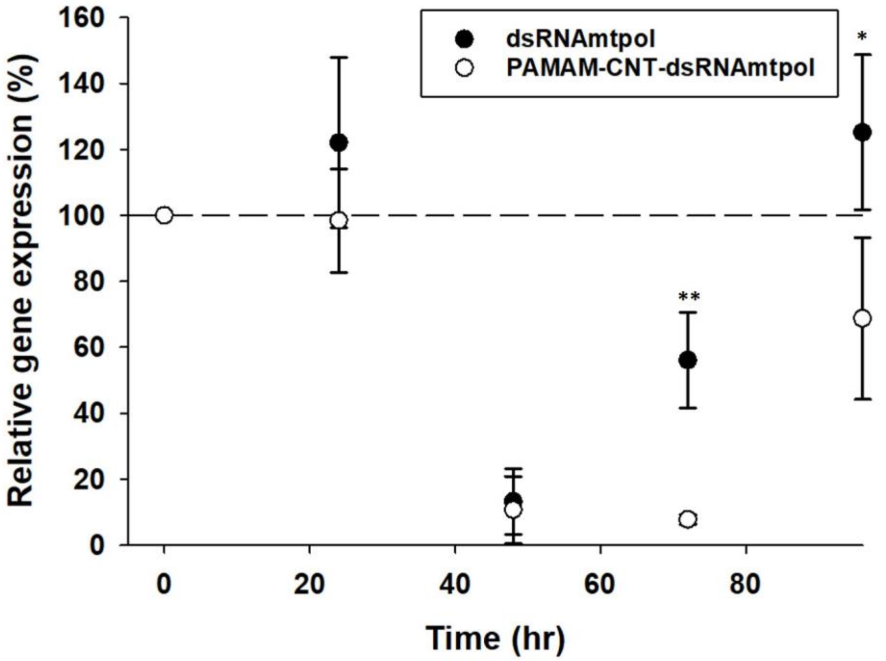

Figure S1. Relative normalised gene expression of targets after injection of PAMAM-CNTs, dsRNA or PAMAM-CNT-dsRNA complexes over 96 hr timecourse. mtpol expression was significantly decreased following injection of naked $dsRNA_{mtpol}$ at at 48h (P<0.001) and 72h (P<0.05) post-injection, recovering to control levels at 96h. mtpol expression was also significantly reduced in PAMAM-CNT-$dsRNA_{mtpol}$ injected larvae at 48h (P<0.001), 72h (P<0.001) and 96h (P<0.05). Expression was significantly lower in PAMAM-CNT-$dsRNA_{mtpol}$ compared to $dsRNA_{mtpol}$ alone at 72h (P<0.01) and 96h (P<0.05) Data are presented as means +/- SEM (n =4 per treatment) as percentage. Asterixes represent significant difference between treatments determined by Student's t-test.
